# Case Report: Genomic insights and personalized treatment in dual primary esophageal squamous cell carcinoma and gastric adenocarcinoma

**DOI:** 10.3389/fonc.2025.1625063

**Published:** 2025-09-02

**Authors:** Chuanwang Miao, Yaning Guo, Huili Yang, Shan Zhu, Yuanji Chen, Wei Zhao, Xudong Hu

**Affiliations:** ^1^ Department of Radiation Oncology, Shandong Cancer Hospital and Institute, Shandong First Medical University and Shandong Academy of Medical Sciences, Jinan, Shandong, China; ^2^ Department of Oncology, Binzhou Central Hospital, Binzhou Central Hospital Affiliated to Binzhou Medical University, Binzhou, Shandong, China; ^3^ Department of Radiation Oncology, Shandong Provincial Ear, Nose, and Throat Hospital, Shandong University, Jinan, China

**Keywords:** dual primary cancer, esophageal squamous cell carcinoma, gastric adenocarcinoma, next generation sequencing, chromosomal alterations

## Abstract

Dual primary malignancies in the upper gastrointestinal tract are rare and pose diagnostic and therapeutic challenges. This study reports a case of synchronous esophageal squamous cell carcinoma (ESCC) and gastric adenocarcinoma (GAC), highlighting the role of genetic profiling in personalized treatment. A 78-year-old female patient was diagnosed with stage IIB ESCC and stage IIIA GAC. Due to surgical ineligibility, she received chemoradiotherapy and immunotherapy. Next-generation sequencing (NGS) was performed to identify potential genetic drivers. Genetic analysis revealed common chromosomal amplifications on 19p and 21q but no shared driver mutations, suggesting independent tumor origins. ESCC exhibited amplifications of MCL1, RECQL4, NKX2-1, PARP10, RSPO1, MUCL, and WTIP, while GAC showed deletions of APC and PRKG1, along with amplifications of ARRDC1 and NRARP. The patient achieved stable disease without recurrence following chemoradiotherapy and Sintilimab immunotherapy. This case underscores the role of genetic alterations in dual primary cancers and demonstrates the feasibility of precision treatment.

## 1 Introduction

Esophageal cancer and gastric cancer are two major malignancies of the digestive tract, which pose a significant threat to human health and are ranked among the leading causes of cancer incidence and mortality worldwide (1, 2). According to literature reports, the probability of esophageal cancer patients developing other malignant tumors ranges from 8.3% to 12.6%, with the occurrence of gastric cancer being relatively rare, at only 0.8% to 1.5% (3, 4). The occurrence of dual primary cancers involving esophageal squamous cell carcinoma (ESCC) and gastric antrum adenocarcinoma is extremely rare. Moreover, these two cancers exhibit significant differences in their pathological types, pathogenesis, and treatment strategies, making it clinically challenging to distinguish them from metastatic cancers (5). In recent years, with the continuous advancement of genetic testing technologies, the molecular biological research on dual primary malignancies has gradually deepened. Previous studies have shown that specific chromosomal and genetic mutations may play an important role in the development of dual primary cancers (6). Furthermore, there are notable differences in the pathogenesis of esophageal squamous cell carcinoma and gastric antrum adenocarcinoma (7). For example, the amplification of chromosome 19p in ESCC may promote tumor invasiveness through the RAS signaling pathway (8), while in gastric antrum adenocarcinoma, the amplification of 19p is closely related to the regulation of the cell cycle (9). At present, research on esophagus-gastric dual primary cancers mainly focuses on diagnosis and treatment (10, 11), while studies on the molecular mechanisms of their development remain limited. In addition, due to the complexity of upper gastrointestinal reconstruction, surgical treatment for patients with dual primary cancers is less feasible (12). Furthermore, there are no specific treatment guidelines for these patients. Currently, a combination of radiotherapy, chemotherapy, and immunotherapy is considered the mainstream treatment strategy. However, whether common chromosomal or gene mutations found in genetic testing serve as shared driving factors requires further case studies and molecular mechanism research to provide additional evidence. This case report details a 78-year-old female patient with esophageal-gastric dual primary cancers who received sequential radiotherapy combined with immunotherapy, exploring the relationship between chromosomal variations and the pathogenesis of dual primary cancers, as well as personalized treatment plans, offering new insights for the diagnosis and treatment of such cases.

## 2 Case description

### 2.1 Patient information

A 78-year-old female patient with no history of smoking or alcohol consumption and reported good overall health presented with complaints of pain while eating on March 23, 2022. Esophagogastroduodenoscopy (EGD) and biopsy revealed esophageal squamous cell carcinoma (ESCC) located 29–34 cm from the incisors and gastric antrum adenocarcinoma. No obvious abnormalities were found in the physical examination and laboratory tests at admission (detailed results available in Supplementary, **Supplementary Table 5**), suggesting that the patient can proceed with follow-up treatment.

### 2.2 Diagnostic assessment

On March 25, 2022, esophagogastroduodenoscopy (EGD) demonstrated suspicious lesions located 29–34 cm from the incisors in the esophagus and a mass in the gastric antrum. Biopsies confirmed esophageal squamous cell carcinoma (ESCC) and gastric antrum adenocarcinoma (GAC).

**Figure 1:**
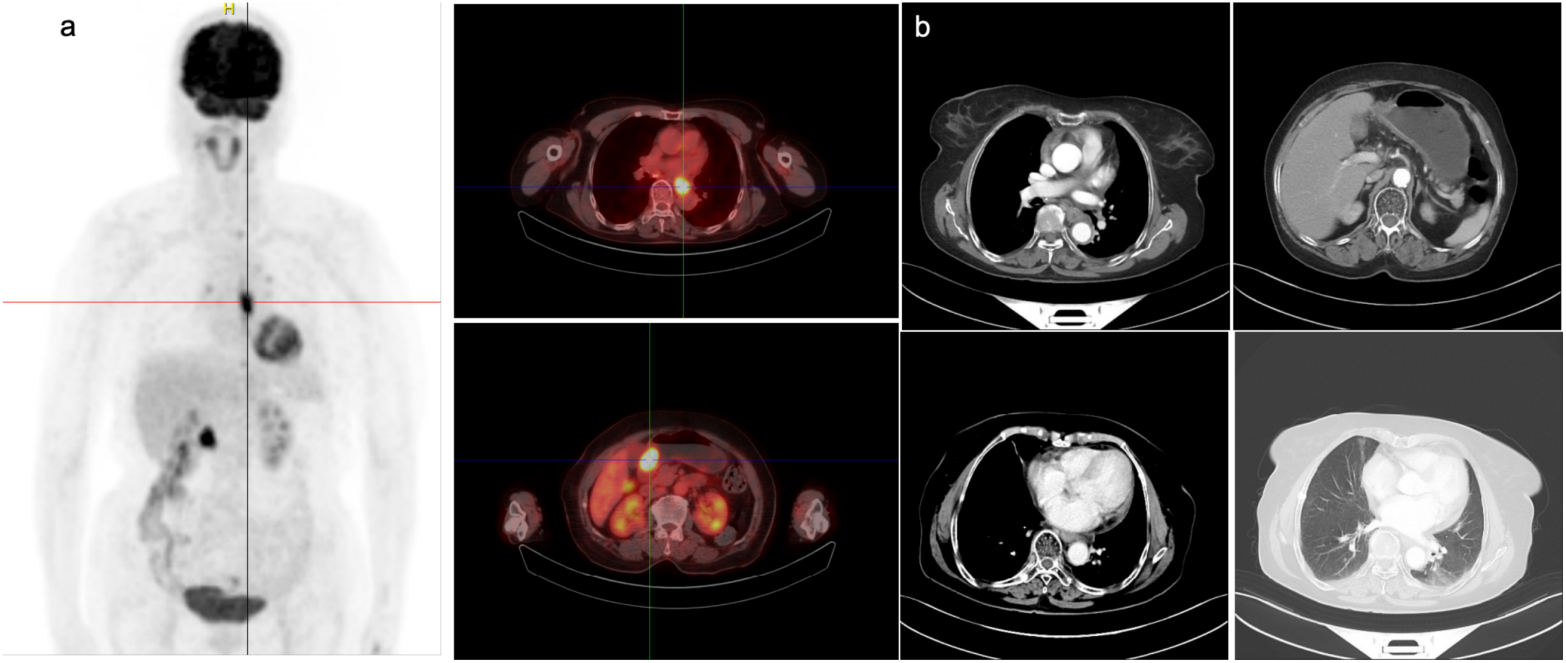
Imaging findings of the patient before and after admission. **(a)** PET-CT imaging on March 31, 2022, showed localized thickening of the mid-thoracic esophageal wall with a maximum SUV of 13.4. Slightly increased uptake was observed at the proximal and distal ends of the esophagus, as well as near the gastroesophageal junction, with a maximum SUV of 4.0. Thickening of the gastric antrum wall was also noted, with a maximum SUV of 15.1. **(b)** CT scan on September 21, 2022, showing no significant change in the esophageal cancer after treatment compared to the previous scan.

**Figure 2:**
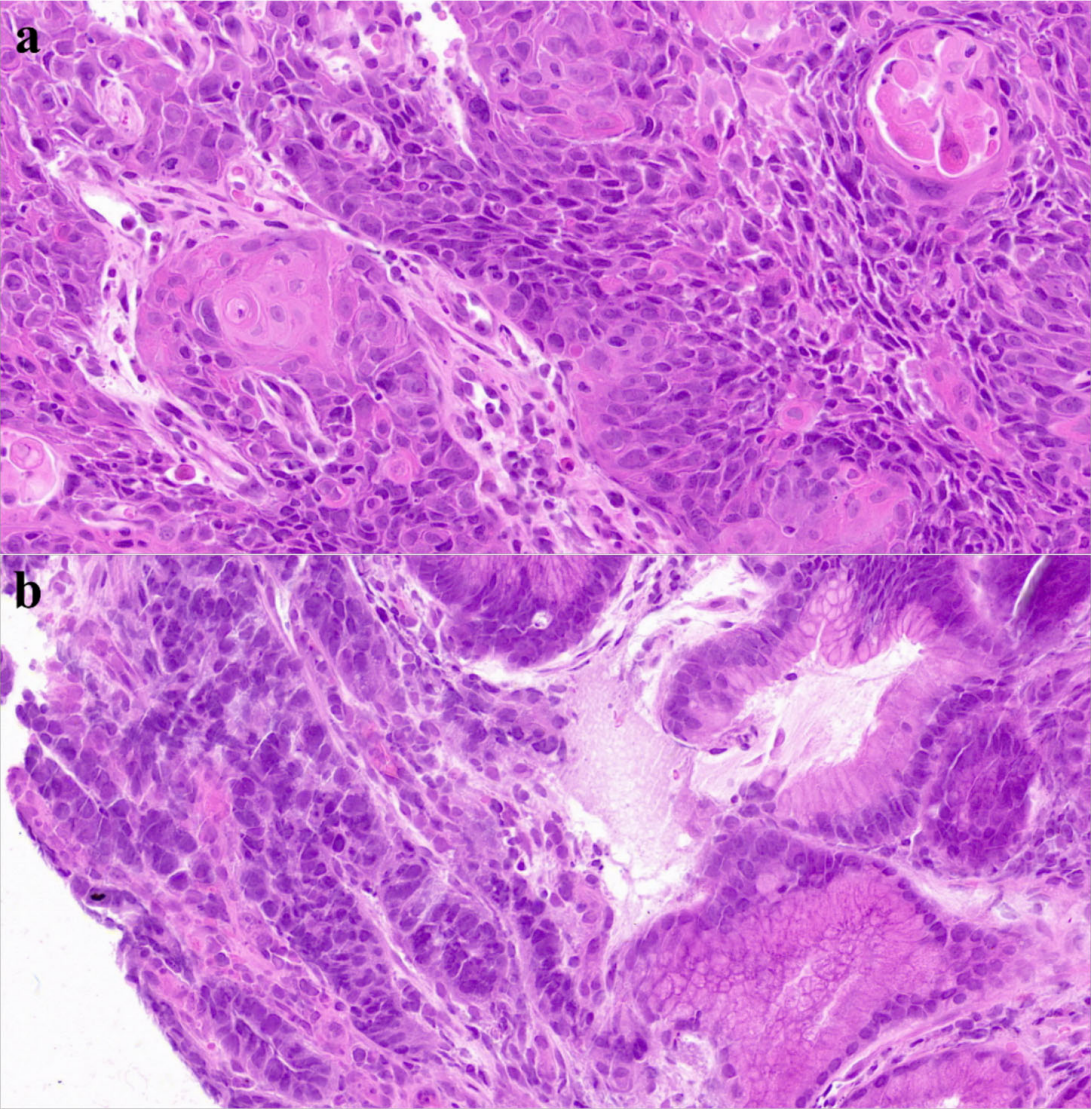
H&E-stained slides of esophageal and gastric carcinoma. **(a)** Histopathological Manifestations of Esophageal Squamous Cell Carcinoma on HE Staining: Atypical Squamous Cell Nests, Keratin Pearls, and Stromal Invasion. **(b)** Histopathological Manifestations of Gastric Adenocarcinoma on HE Staining: irregular glandular structures with distorted architecture, nuclear atypia showing enlarged hyperchromatic nuclei, loss of polarity, frequent mitoses, and evident stromal desmoplasia.

On March 31, 2022, PET-CT (**Figure 1a**) showed hypermetabolic foci in the mid-esophagus (SUVmax=13.4), gastric antrum (SUVmax=15.1), and regional lymph nodes (SUVmax=4.0), confirming dual primaries without metastasis. Histopathology revealed invasive ESCC with keratin pearls (**Figure 2a**) and GAC with desmoplastic stroma (**Figure 2b**).

### 2.3 Therapeutic intervention

**Figure 3:**
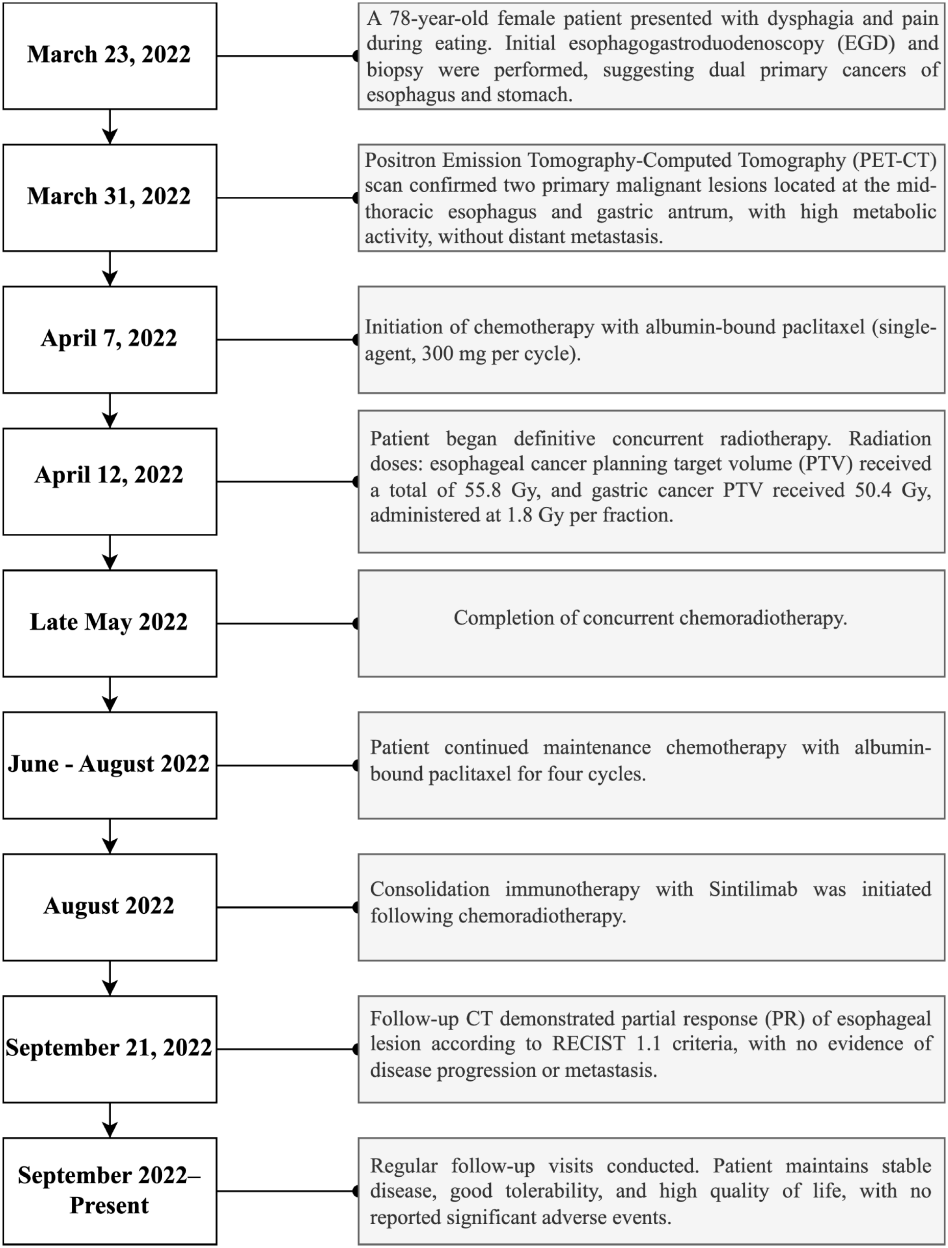
Timeline summarizing key clinical events, diagnostic assessments, therapeutic interventions, and follow-up outcomes during patient management.

Multidisciplinary therapy included albumin-bound paclitaxel (300 mg, initiated April 7) and image-guided radiotherapy (esophageal PTV: 55.8 Gy; gastric PTV: 50.4 Gy, 1.8 Gy/fraction from April 12) with supportive care (**Supplementary Table 6**). Post-radiation chemotherapy (4 cycles) and Sintilimab consolidation achieved partial response (RECIST 1.1, **Figure 1b**) and stable disease. Next-generation sequencing (NGS) was performed on pathological samples to identify driver genetic/chromosomal alterations in esophageal and gastric cancers. Follow-up labs remained normal (**Supplementary Tables 7, 8**), and the patient’s treatment process is shown in **Figure 3**. The patient maintains good quality of life with no major adverse events.

Based on the histological findings, along with PET-CT imaging and clinical presentation, the final diagnosis was esophageal squamous cell carcinoma (T3N0M0, stage IIB) and gastric antrum adenocarcinoma (T3N2M0, stage IIIA).

## 3 Sample processing and genetic testing

Initially, for the purpose of elucidating the pathogenesis of the patient’s esophageal squamous cell carcinoma (ESCC) and gastric antrum adenocarcinoma, tumor tissue samples were collected before treatment and underwent genetic testing. After quality control and cleaning procedures to remove low-quality reads and bases, the reliability of the data was ensured (**Supplementary Table 1** in **Supplementary Material**).

Subsequently, the patient’s tumor tissue samples were thoroughly analyzed for chromosomal and genetic variations, with a focus on detecting mutations associated with cancer, including single nucleotide variants (SNVs) and copy number variations (CNVs). To ensure the reliability and clinical relevance of the data, a strict quality control standard was applied during the selection process. Specific selection criteria are shown in **Supplementary Tables 2** in **Supplementary Material**.

The sequencing results showed that after quality control screening, key chromosomal and genetic variations were successfully identified in both esophageal squamous cell carcinoma (ESCC) and gastric antrum adenocarcinoma (GAC) tissues. Common amplifications were observed on chromosomes 19p and 21q in both cancer tissues, while differential amplifications and deletions were noted on other chromosomes (Shown in **Supplementary Tables 3, 4** in the **Supplementary Material**). The specific gene mutations in the two tumor tissues are shown in **Supplementary Tables S9**, **S10** in **Supplementary Material**. But no significant common gene mutations were found between the two cancers.

Although no common pathogenic gene mutations were found in the esophageal and gastric cancer tissues at the genetic level.

## 4 Discussion

**Figure 4:**
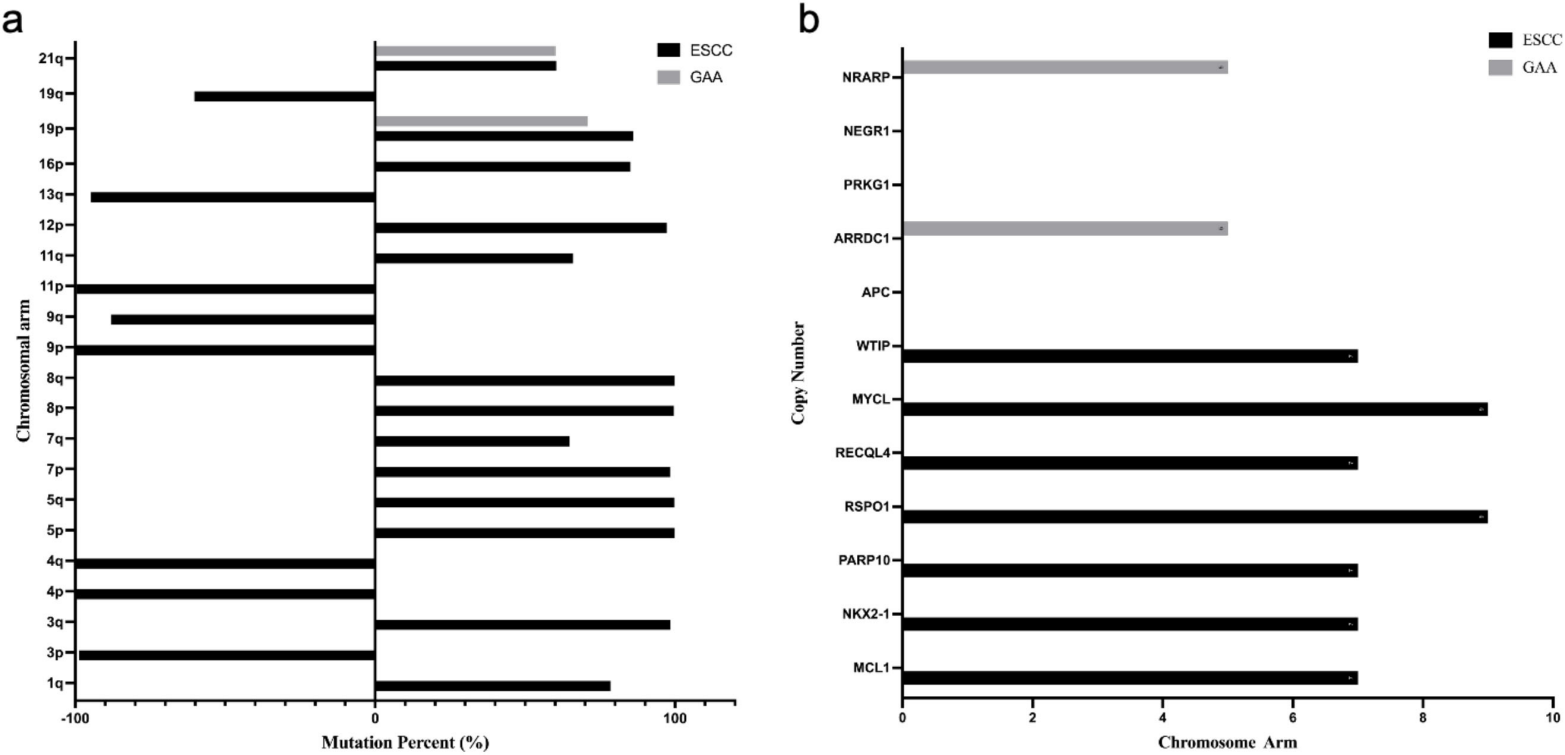
Chromosomal variations and gene mutation - copy number in esophageal and gastric tumor tissues. **(a)** Chromosomal Variations in Esophageal Squamous Cell Carcinoma and Gastric Antrum Adenocarcinoma Tissues:The right side of the Y-axis represents amplification, and the left side represents deletion. **(b)** Gene Mutation Copy Number Distribution in Tumor Tissues: The copy number of NEGR1, PRKG1, and APC is 0.

In this case, genetic testing revealed common chromosomal amplifications on 19p and 21q in both ESCC and GAC tissues (shown in **Figure 4a**). Chromosome 19p amplification has previously been associated with increased tumor invasiveness through activation of the RAS signaling pathway in esophageal cancer and with regulation of cell cycle progression in gastric cancer. Although amplification on chromosome 21q is less frequently reported, it may significantly enhance tumor aggressiveness. Notably, RUNX1 (21q22.12) amplification is linked with increased invasiveness and metastatic potential in esophageal cancer, consistent with patterns of chromosomal instability (CIN). In gastric cancer, amplification of ETS2 (21q22.3) promotes tumor proliferation by activating the RAS/MAPK pathway, and it has also been associated with chemotherapy resistance. However, the predominant type of chromosomal alteration on 21q reported in literature remains deletion, with amplification primarily observed in advanced or aggressive cases.

Gene alterations were identified in each tumor, which are of significant clinical and research importance (Show in **Figure 4b**). In ESCC tissue, amplification of genes such as WTIP and MCL1 (chromosome 19) may enhance tumor invasiveness and anti-apoptotic capacities, potentially influencing tumor progression and therapeutic response (13, 14). In GAC tissue, the deletion of APC likely activates the Wnt signaling pathway, contributing to poor prognosis, while amplification of ARRDC1 may increase invasiveness through autophagy-related mechanisms (15, 16).

Due to tumor complexity and advanced age, surgery was unsuitable. Instead, synchronous chemoradiotherapy and immunotherapy reduced tumor burden and facilitated immune clearance of residual disease.

Concurrent chemoradiotherapy is an effective treatment for esophageal cancer, showing significant efficacy and survival benefits, particularly in locally advanced patients (17, 18). It also demonstrates some efficacy in patients with synchronous esophagus-gastric dual primary cancers (19). The weekly regimen of cisplatin and albumin-bound paclitaxel combined with radiotherapy has been shown to be safe with manageable toxicity and good antitumor activity in patients with locally advanced esophageal squamous cell carcinoma (20). However, this regimen is rarely used in early-stage patients, and therefore, prognostic information is lacking. In this case, the regimen was successfully applied, significantly reducing the tumor burden and creating optimal conditions for subsequent immunotherapy to enhance immune system activation and tumor clearance.

In elderly patients, the tolerability and safety of immunotherapy are critical. In this case, immunotherapy with Sintilimab significantly improved the patient’s condition while avoiding severe treatment-related toxicities, which aligns with the results of the ORIENT-15 study (21, 22). Furthermore, Sintilimab has also demonstrated promising efficacy in perioperative treatment for locally advanced gastric antrum adenocarcinoma (23). The favorable clinical outcome highlights the efficacy of chemoradiotherapy combined with immunotherapy as a personalized approach for elderly patients with dual primary cancers lacking clear genetic targets.

## 5 Conclusions

This case reveals shared chromosomal mutations (19p/21q) in esophageal squamous cell carcinoma and gastric adenocarcinoma, offering initial insights into dual primary cancer pathogenesis. While no common driver genes were identified, tumor-specific mutations may inform future targeted therapies. For non-metastatic dual cancers, functionally-guided radical chemoradiotherapy was successfully implemented, using metabolic activity-based radiation tailored to tumor heterogeneity and tolerability. This precision approach achieved partial response and stable disease (RECIST 1.1), providing critical clinical evidence for optimizing dual cancer management. The findings underscore the importance of genomic profiling and adaptive radiotherapy in advancing pathogenesis research and therapeutic strategies for esophagus-gastric dual malignancies.

## Data Availability

All data from this literature are presented in the submitted supplementary files.
