# Supplementary material for: Case Report: Genomic insights and personalized treatment in dual primary esophageal squamous cell carcinoma and gastric adenocarcinoma
Source: Front Oncol. 2025 Sep 2;15:1625063. doi: 10.3389/fonc.2025.1625063 (PMC12436115; doi:10.3389/fonc.2025.1625063)
Supplement: Supplementary file 1 [file Table1.docx]

Supplementary Material

Sample Processing and Genetic Testing:

Pre-treatment tumor tissues from esophageal squamous cell carcinoma (ESCC) and gastric adenocarcinoma (GAC) underwent next-generation sequencing (NGS) to identify genomic drivers. After quality control (QC) filtering low-quality reads (Supplementary **Table 1**), chromosomal and genetic alterations—including single nucleotide variants (SNVs) and copy number variations (CNVs)—were analyzed using stringent clinical relevance criteria (Supplementary **Table 2**). Sequencing results are presented in **Tables 3-4** and **Table 9-10** of the Supplementary file.

| **Sample** | **FB22630156 - A3ACF91RNF1 - KBV5** | **BC22630155 - B1BCF91RNF1 - KBV5** | **FB22630157 - A3ACF91RNF1 - KBV5** |
| --- | --- | --- | --- |
| TYPE | Tumor | Normal | Tumor |
| FAIL | DedupDepth Fail | - | - |
| PF_READS | 12409892 | 113645595 | 31093175 |
| CLEAN_READS | 11904286 | 109456334 | 30023606 |
| RATIO_OF_READS (%) | 95.93 | 96.31 | 96.56 |
| PF_BASES | 3747 | 34320 | 9390 |
| CLEAN_BASES | 3313 | 32364 | 8743 |
| RATIO_OF_BASES (%) | 88.42 | 94.3 | 93.11 |
| INSERT_SIZE | 209 | 328 | 280 |
| MAPQ20(%) | 92.13 | 96 | 94.71 |
| DUPLICATE (%) | 31.74 | 38.2 | 41.27 |
| ON_TARGET_CORE (%) | 46.74 | 44.81 | 47.17 |
| ON_TARGET_EXT (%) | 71.9 | 74.17 | 75.44 |
| MEAN_DEPTH | 46.74 | 422.22 | 121.26 |
| MEDIAN_DEPTH | 35 | 391 | 106 |
| 1X_COVERAGE (%) | 98.59 | 98.87 | 98.84 |
| 10X_COVERAGE (%) | 89.94 | 98.58 | 98.39 |
| 20X_COVERAGE (%) | 72.86 | 98.46 | 97.8 |
| 50X_COVERAGE (%) | 35.49 | 98.14 | 88.34 |
| CONTAMINATION | 0.0115 | 0.0003 | 0.0017 |

Supplementary Table 1：Sequencing Data Quality Assessment Statistics

Sample: Different tissue samples: FB22630156-A3ACF91RNF1-KBV5 and FB22630157-A3ACF91RNF1-KBV5 representing tumor tissues from esophageal cancer and gastric antrum adenocarcinoma; BC22630155-B1BCF91RNF1-KBV5 representing normal tissue

PF_READS: Number of raw reads

CLEAN_READS: Number of cleaned reads

RATIO_OF_READS (%): Proportion of reads

PF_BASES: Number of raw bases

CLEAN_BASES: Number of cleaned bases

RATIO_OF_BASES (%): Proportion of bases

INSERT_SIZE: Insert fragment size

MAPQ20(%): Proportion of reads with a mapping quality score greater than 20

DUPLICATE (%): Proportion of duplicate reads

ON_TARGET_CORE (%): Proportion of coverage in the core target region

ON_TARGET_EXT (%): Proportion of coverage in the extended target region

MEAN_DEPTH: Average depth

MEDIAN_DEPTH: Median depth

1X_COVERAGE (%): 1x coverage percentage

10X_COVERAGE (%): 10x coverage percentage

20X_COVERAGE (%): 20x coverage percentage

50X_COVERAGE (%): 50x coverage percentage

CONTAMINATION: Contamination proportion

| Mutation Type | Selection Criteria |
| --- | --- |
| Single Nucleotide Variants (SNV) | Select key mutations in genes that can potentially impact protein function, such as insertions, deletions, nonsense mutations, and splice site mutations, limited to protein-coding genes. |
| Copy Number Variations (CNV) | **Color intensity level**: Identifies deep amplifications or deletions, and the impact on the image segment length exceeds 60% of the tissue region. |
|  | **Base-level**: Focuses on hot spot genes' deep amplification or deletion. |
| Allele Frequency Filtering | Exclude mutations in population databases (1kg, ExAC, gnomAD) with a frequency greater than 1%. Focus on low-frequency mutations. |
| Outlier Allele Frequency | Only analyze variants with an allele frequency greater than 2% in the sample to avoid false positives or low-frequency variants. |

Supplementary Table 2: Selection Criteria

| Arm | Type | Percent |
| --- | --- | --- |
| 1q | Amplification | 78.51% |
| 3p | Deletion | 98.79% |
| 3q | Amplification | 98.46% |
| 4p | Deletion | 99.89% |
| 4q | Deletion | 99.85% |
| 5p | Amplification | 99.81% |
| 5q | Amplification | 99.74% |
| 7p | Amplification | 98.34% |
| 7q | Amplification | 64.84% |
| 8p | Amplification | 99.67% |
| 8q | Amplification | 99.81% |
| 9p | Deletion | 99.89% |
| 9q | Deletion | 87.97% |
| 11p | Deletion | 99.38% |
| 11q | Amplification | 66.04% |
| 12p | Amplification | 97.22% |
| 13q | Deletion | 94.72% |
| 16p | Amplification | 85.12% |
| 19p | Amplification | 86.08% |
| 19q | Deletion | 60.26% |
| 21q | Amplification | 60.42% |

Supplementary Table 3：Chromosomal Copy Number Variation Statistics in Esophageal Squamous Cell Carcinoma Samples; Arm: Chromosomal arm where the mutation occurs; Type: Mutation type; Percent: Incidence rate of the corresponding mutation

| **Arm** | **Type** | **Percent** |
| --- | --- | --- |
| 19p | Amplification | 70.92% |
| 21q | Amplification | 60.08% |

Supplementary Table 4: Chromosomal Copy Number Variation Statistics in Gastric Cancer Samples; Arm: Chromosomal arm where the mutation occurs; Type: Mutation type; Percent: Incidence rate of the corresponding mutation

| **Item** | **Value** | **Reference Range** | **Abnormality** |
| --- | --- | --- | --- |
| White Blood Cell Count | 5.26×10⁹/L | 3.5–9.5×10⁹/L | Normal |
| Red Blood Cell Count | 4.23×10¹²/L | 3.8–5.1×10¹²/L | Normal |
| Hemoglobin | 106 g/L | 115–150 g/L | ↓ Decreased |
| Platelet Count | 316×10⁹/L | 125–350×10⁹/L | Normal |
| Neutrophil Count | 3.08×10⁹/L | 1.8–6.3×10⁹/L | Normal |
| Monocyte Count | 0.41×10⁹/L | 0.1–0.6×10⁹/L | Normal |
| Albumin | 39.8 g/L | 40–55 g/L | ↓ Decreased |
| Prealbumin | 0.158 g/L | 0.2–0.4 g/L | ↓ Decreased |
| Uric Acid | 152.0 μmol/L | 210–420 μmol/L | ↓ Decreased |
| Alkaline Phosphatase | 129 U/L | 40–125 U/L | ↑ Increased |
| High-density Lipoprotein Cholesterol (HDL-C) | 0.90 mmol/L | ≥1.04 mmol/L | ↓ Decreased |
| Low-density Lipoprotein Cholesterol (LDL-C) | 3.43 mmol/L | <3.37 mmol/L | ↑ Increased |
| Carcinoembryonic Antigen (CEA) | 2.96 ng/mL | 0–5 ng/mL | ↑ Increased |
| Neuron-Specific Enolase (NSE) | 14.80 ng/mL | 0–16.3 ng/mL | Normal |
| Cytokeratin-19 Fragment (CYFRA21-1) | 2.38 ng/mL | 0–3.3 ng/mL | ↑ Increased |
| Progastrin-Releasing Peptide (ProGRP) | 54.29 pg/mL | 0–65 pg/mL | Normal |
| Carbohydrate Antigen CA19-9 | 74.85 U/mL | 0–37 U/mL | ↑ Increased |

Supplementary Table 5：Initial laboratory test results at admission on March 30, 2022, including complete blood count, liver function, lipid profile, and tumor markers. Arrows indicate values outside the normal reference range.

| **Item** | **Value** | **Reference Range** | **Abnormality** |
| --- | --- | --- | --- |
| Red Blood Cell Count | 4.07×10¹²/L | 3.8-5.1×10¹²/L | Normal |
| Hemoglobin | 103 g/L | 115-150 g/L | ↓ Decreased |
| Platelet Count | 248×10⁹/L | 125-350×10⁹/L | Normal |
| Neutrophil Count | 1.25×10⁹/L | 1.8-6.3×10⁹/L | ↓ Decreased |
| Monocyte Count | 0.14×10⁹/L | 0.1-0.6×10⁹/L | Normal |
| Carcinoembryonic Antigen (CEA) | 8.52 ng/mL | 0–5 ng/mL | Normal |
| Neuron-Specific Enolase (NSE) | 14.71 ng/mL | 0–16.3 ng/mL | Normal |
| Cytokeratin-19 Fragment (CYFRA21-1) | 7.23 ng/mL | 0–3.3 ng/mL | Normal |
| Progastrin-Releasing Peptide (ProGRP) | 41.96 pg/mL | 0–65 pg/mL | Normal |
| Carbohydrate Antigen CA19-9 | 7 U/mL | 0–37 U/mL | Normal |

Supplementary Table 6：Laboratory test results on April 11, 2022, demonstrating decreased hemoglobin and neutrophil count after initial treatments.

| **Item** | **Value** | **Reference Range** | **Abnormality** |
| --- | --- | --- | --- |
| Red Blood Cell Count | 3.97×10¹²/L | 3.8–5.1×10¹²/L | Normal |
| Hemoglobin | 108 g/L | 115–150 g/L | ↓ Decreased |
| Platelet Count | 245×10⁹/L | 125–350×10⁹/L | Normal |
| Neutrophil Count | 1.78×10⁹/L | 1.8–6.3×10⁹/L | ↓ Decreased |
| Monocyte Count | 0.29×10⁹/L | 0.1–0.6×10⁹/L | Normal |

Supplementary Table 7：Laboratory test results on June 14, 2022, showing persistent reductions in hemoglobin and neutrophil count during ongoing radiotherapy.

| **Item** | **Value** | **Reference Range** | **Abnormality** |
| --- | --- | --- | --- |
| Red Blood Cell Count | 3.85×10¹²/L | 3.8–5.1×10¹²/L | Normal |
| Hemoglobin | 98 g/L | 115–150 g/L | ↓ Decreased |
| Platelet Count | 247×10⁹/L | 125–350×10⁹/L | Normal |
| Neutrophil Count | 1.47×10⁹/L | 1.8–6.3×10⁹/L | ↓ Decreased |
| Monocyte Count | 0.51×10⁹/L | 0.1–0.6×10⁹/L | Normal |
| Lymphocyte Count | 1.38×10⁹/L | 1.1–3.2×10⁹/L | Normal |
| Carcinoembryonic Antigen (CEA) | 2.77 ng/mL | 0–5 ng/mL | Normal |
| Neuron-Specific Enolase (NSE) | 12.00 ng/mL | 0–16.3 ng/mL | Normal |
| Cytokeratin-19 Fragment (CYFRA21-1) | 1.28 ng/mL | 0–3.3 ng/mL | Normal |
| Progastrin-Releasing Peptide (ProGRP) | 25.70 pg/mL | 0–65 pg/mL | Normal |
| Carbohydrate Antigen CA19-9 | 11.80 U/mL | 0–37 U/mL | Normal |

Supplementary Table 8：Follow-up laboratory test results on September 21, 2022, reflecting continued anemia and neutropenia, with normal levels of tumor markers.

| **Chrom** | **Gene Name** | **Copy level** | **Copy Number** | **CNV Type** | **Gene Type** |
| --- | --- | --- | --- | --- | --- |
| 1 | MCL1 | 2 | 7 | Deep_Amplification | hotgene |
| 14 | NKX2-1 | 2 | 7 | Deep_Amplification | hotgene |
| 8 | PARP10 | 2 | 7 | Deep_Amplification | hotgene |
| 1 | RSPO1 | 2 | 9 | Deep_Amplification | hotgene |
| 8 | RECQL4 | 2 | 7 | Deep_Amplification | hotgene |
| 1 | MYCL | 2 | 9 | Deep_Amplification | hotgene |
| 19 | WTIP | 2 | 7 | Deep_Amplification | hotgene |

Table 9: Analysis of Key Gene Amplifications and Deletions in Esophageal Cancer Samples: Chrom: Chromosome number where the mutation is located； Copy Level: Copy number variation level; Copy Number: Actual copy number; CNV Type: Type of copy number variation.

| **Chrom** | **Gene Name** | **Copy level** | **Copy Number** | **CNV Type** | **Gene Type** |
| --- | --- | --- | --- | --- | --- |
| 5 | APC | -2 | 0 | Deep_Deletion | hotgene |
| 9 | ARRDC1 | 2 | 5 | Deep_Amplification | hotgene |
| 10 | PRKG1 | -2 | 0 | Deep_Deletion | hotgene |
| 1 | NEGR1 | -2 | 0 | Deep_Deletion | hotgene |
| 9 | NRARP | 2 | 5 | Deep_Amplification | hotgene |

Table10: Analysis of Key Gene Amplifications and Deletions in Gastric Antrum Adenocarcinoma Samples: Chrom: Chromosome number where the mutation is located； Copy Level: Copy number variation level; Copy Number: Actual copy number; CNV Type: Type of copy number variation.
